# Development of Conductive Gelatine-Methacrylate Inks for Two-Photon Polymerisation

**DOI:** 10.3390/polym13071038

**Published:** 2021-03-26

**Authors:** Paola Sanjuan-Alberte, Jayasheelan Vaithilingam, Jonathan C. Moore, Ricky D. Wildman, Christopher J. Tuck, Morgan R. Alexander, Richard J. M. Hague, Frankie J. Rawson

**Affiliations:** 1 Regenerative Medicine and Cellular Therapies, Biodiscovery Institute, School of Pharmacy, University of Nottingham, University Park, Nottingham NG7 2RD, UK; 2 Department of Bioengineering and Institute for Bioengineering and Biosciences, Instituto Superior Técnico, Universidade de Lisboa, Av. Rovisco Pais, 1049-001 Lisboa, Portugal; 3 Centre for Additive Manufacturing, Faculty of Engineering, University of Nottingham, University Park, Nottingham NG7 2RD, UK; ezajv4@exmail.nottingham.ac.uk (J.V.); ezzrdw@exmail.nottingham.ac.uk (R.D.W.); ezzcjt@exmail.nottingham.ac.uk (C.J.T.); ezzrjh@exmail.nottingham.ac.uk (R.J.M.H.); 4 School of Chemistry, University of Nottingham, University Park, Nottingham NG7 2RD, UK; j.moore@nottingham.ac.uk; 5 Advanced Materials and Healthcare Technologies, School of Pharmacy, University of Nottingham, University Park, Nottingham NG7 2RD, UK; pazma1@exmail.nottingham.ac.uk

**Keywords:** conductive hydrogels, GelMa, carbon nanotubes, two-photon polymerization, nano-fabrication

## Abstract

Conductive hydrogel-based materials are attracting considerable interest for bioelectronic applications due to their ability to act as more compatible soft interfaces between biological and electrical systems. Despite significant advances that are being achieved in the manufacture of hydrogels, precise control over the topographies and architectures remains challenging. In this work, we present for the first time a strategy to manufacture structures with resolutions in the micro-/nanoscale based on hydrogels with enhanced electrical properties. Gelatine methacrylate (GelMa)-based inks were formulated for two-photon polymerisation (2PP). The electrical properties of this material were improved, compared to pristine GelMa, by dispersion of multi-walled carbon nanotubes (MWCNTs) acting as conductive nanofillers, which was confirmed by electrochemical impedance spectroscopy and cyclic voltammetry. This material was also confirmed to support human induced pluripotent stem cell-derived cardiomyocyte (hPSC-CMs) viability and growth. Ultra-thin film structures of 10 µm thickness and scaffolds were manufactured by 2PP, demonstrating the potential of this method in areas spanning tissue engineering and bioelectronics. Though further developments in the instrumentation are required to manufacture more complex structures, this work presents an innovative approach to the manufacture of conductive hydrogels in extremely low resolution.

## 1. Introduction

Conductive hydrogels are gaining significant interest from the bioelectronics community as they can act as soft interfaces between biological and electronic systems, offering an alternative to traditional inorganic materials [1].

Hydrogels are highly hydrated polymeric networks with water contents similar to that of soft tissues [2]. To mimic in vivo conditions more accurately, it is common to develop three-dimensional (3D) cellular scaffolds based on hydrogels for regenerative medicine and tissue engineering applications. This is due to their biocompatibility and because they can create porous, soft and elastic interfaces [3]. Furthermore, the mechanical properties of hydrogels are comparable to biological tissues. Some examples of biomimetic hydrogel scaffolds with therapeutic applications include alginate and collagen-based hydrogels for applications in wound healing, cartilage repair, bone regeneration or drug delivery [4,5].

Strategies to enhance the electrical properties of hydrogels include the integration of conductive polymers and conductive nano-fillers into the hydrogel matrix [6,7]. Some examples include the incorporation of poly(3,4-ethylenedioxythiophene) (PEDOT): polystyrene sulfonate (PSS) to polyethylene glycol diacrylate (PEGDA) for neural tissue engineering applications [8] and the dispersion of gold nanorods into gelatine for the engineering of cardiac constructs [9]. The overall conductivity of these materials combines the electronic functionality of the conductive elements and the ionic contribution from inter- and intra-chain charge transfer throughout the matrix/networks [10].

Cells can respond to different types of external stimuli, including the chemical composition of their surroundings [11], stiffness [12], topography [13] or electrical stimulation [14]. Therefore, controlling and combining these different aspects is important to create biomimetic architectures with the capacity to promote tissue organisation and response after injury. Additive manufacturing (AM) techniques, notably inkjet-based 3D printing, extrusion printing and vat polymerisation AM techniques offer the possibility of creating structures that can be easily tuned and fitted for a specific application [15,16,17]. In particular, two-photon polymerisation (2PP) holds great potential in the engineering of the local cellular microenvironments due to the high resolution (<100 nm) of printed structures that can be achieved with this technique [18,19].

Photo-polymerisation of hydrogels leads to the formation of a polymeric network with high water content [20]. Photopolymerisable formulations often consist of a photoinitiator, cross-linkers and a solvent [20]. Most of the hydrogel inks developed for 2PP are based on poly(ethylene glycol) diacrylate (PEGDA) [21,22,23], bovine serum albumin (BSA) [24] or gelatine-methacrylate (GelMa) [25,26,27]. Despite the biochemical and biomechanical control that can be achieved using these materials, there are no known reports of conductive gels developed for 2PP being investigated for increased electrical properties.

In previous work, we have demonstrated the ability of multi-walled carbon nanotubes (MWNCTs) to act as conductive nano-fillers of 2PP-processed polymeric structures based on pentaerythritol triacrylate (PETrA) [18]. Others have also reported the use of MWCNTs to fabricate 3D conductive structures by 2PP based on thiol-acrylate composites [28] and the acrylate monomer R712 [29,30]. However, this has not been attempted with hydrogels.

For the first time, this work presents a strategy to manufacture 3D microstructures by 2PP with enhanced electrical properties for potential applications in electronics and bioelectronics based on conductive hydrogels. To achieve this, a conductive photocurable ink was formulated by dispersing MWCNTs into a GelMa solution incorporating a water-soluble photoinitiator. Electrical properties of the hydrogels were assessed by impedance spectroscopy and cyclic voltammetry, demonstrating an enhanced electrical conductivity and electron transfer of the MWCNTs-modified GelMa (MWCNTs-GelMa). This material was also compatible with human pluripotent stem cell-derived cardiomyocytes (hPSC-CMs), demonstrating its potential in tissue engineering and regenerative medicine. The last part of the work presented herein was to develop micron-sized biomimetic scaffolds by 2PP using the conductive hydrogel inks.

## 2. Materials and Methods

### 2.1. GelMa Preparation

Gelatine from porcine skin (Sigma Aldrich, St. Louis, MO, USA) was added to warm Dulbecco’s phosphate buffer saline (DPBS) (Thermo Fischer, Waltham, MA, USA) at a concentration of 100 mg mL^−1^ and stirred until fully dissolved at 50 °C. When the gelatine solution was clear, methacrylic anhydride (MAA, Sigma Aldrich) at a concentration of 8% (v/v) was added dropwise. After this, the solution was left stirring for 3 more h at 50 °C. The solution was then diluted 1:5 in DBPS to reach a final gelatine concentration of 20 mg mL^−1^. Gelatine methacrylate (GelMa) solution was placed in 2000 MWCO dialysis tubes (Sigma Aldrich) and dialysed in ultrapure water at 45–50 °C. Water was changed three times a day for 1 week. Once GelMa solution was dialysed, it was placed at −80 °C for 24 h, lyophilised and stored until further used.

### 2.2. Ink Development

Sodium 3,3′-((((1E,1’E)-(2-oxocyclopentane-1,3-diylidene) bis (methanylylidene))bis(4,1-phenylene))bis(methylazanediyl)) dipropanoate (P2CK) was used as photoinitiator. Preparation of P2CK was performed following previous protocols [31]. Lyophilised GelMa was reconstituted in DPBS at a concentration of 5% (*w*/*v*) at 50 °C for 10 min. In solutions containing carboxyl-functionalised multi-walled carbon nanotubes (MWCNTs, OD 15 ± 5 nm, length 1–5 µm, Nano-Lab), a concentration of 5 mg mL^−1^ MWCNTs was used and sonicated for 1 hour, centrifuged at 40 °C for 30 min at 6000 rpm and used as a stock solution. Selected amounts of this solution were added to 5% GelMa solutions containing 0.5% w/v P2CK to make the different inks.

For SEM imaging, materials were lyophilised using the previous procedure, placed in a titanium pin stub with a carbon tab and coated with a thin platinum layer before SEM imaging in a field-emission scanning electron microscope (FEG-SEM JOEL 7100F) in secondary electron mode with an acceleration voltage of 5 kV and a working distance of 10 mm.

For cell tests, GelMa and MWCNTs-GelMa solutions were added to a 96-well plate and UV-cross-linked using a LED lamp (25 mm × 10 mm FireFly UV LED lamp from Phoseon Technology, Hillsboro, OR, USA) with a wavelength of 385 nm for 3 min. This lamp consists of an array of LEDs with a total power density of 4 W cm^−2^. The distance between the LED lamp and the 96-Well plate was 1 cm.

Gels used in subsequent experiments with cells were washed in a 1% antibiotic solution in DBPS (penicillin/streptomycin solution 10,000 units penicillin 10 mg streptomycin/mL; Sigma Aldrich) for 1 h and thoroughly washed in PBS. Samples were sealed with parafilm and stored hydrated in the fridge when not immediately used.

### 2.3. NMR Characterisation

The degree of conversion was quantified by characterising the samples using ^1^H Nuclear Magnetic Resonance at 400 MHz (NMR, DPX UltraShield 400 MHz, Bruker UK ltd, Coventry, UK). A sample of lyophilised gelatine and 2 batches of GelMa (GB1 and GB2) prepared separately were used for this characterisation. Characterisation samples were prepared using deuterium oxide as a solvent. The area under the peak of lysine methylene proton of GelMa (X) and the lysine methylene proton of gelatine (Y) was used to compute the degree of conversion as follows:

(1) $$Degree\;of\;conversion\;\left(DoC\right)=\;\left[1-\;\left(\frac{X}{Y}\right)\right]\times \;100.$$

### 2.4. Electrochemical Characterisation of Hydrogels

Electrochemical impedance spectroscopy was performed on a PGSTAT potentiostat including a FRA32M module (Metrohm Autolab, Utrecht, The Netherlands) and interfaced with a personal computer including the NovaLab software. Briefly, 1 cm diameter gels were photopolymerised in situ and sandwiched between two gold electrodes (Georg-Albert PVD, Silz, Germany) in a two-electrode configuration at room temperature. Frequencies ranging from 10 to 1,000,000 Hz were applied. For this, 1 mg mL^−1^ MWCNTs-GelMa hydrogels were used. Each measurement was performed in triplicate.

Cyclic voltammetry was performed in the aforementioned instrument. Gels were formed in situ on a gold electrode connected to an electrochemical cell and used as a working electrode. A silver/silver chloride electrode was used as a reference electrode and a platinum wire was used to close the circuit. Potentials were applied from 0.8 to −0.2 V at a scan rate of 100 mV s^−1^. Phosphate buffered saline (PBS) and 1 mM potassium hexacyanoferrate (II) (Sigma-Aldrich) in PBS solutions were used as supporting electrolyte and redox-active species, respectively. For this, 1 mg mL^−1^ MWCNTs-GelMa hydrogels were used. Each measurement was performed in triplicate.

### 2.5. hPSC-CMs Differentiation

A REBL-PAT hPSC line was derived from a skin punch biopsy from a male subject. Procedures of isolation, culture, differentiation and dissociation are described elsewhere [32]. Early hPSC-CMs were seeded on the photo-cured gels. Dissociation of cells took place 6–8 weeks after the differentiation process and cells were seeded on hydrogels at an approximate concentration of 2 million cells mL^−1^ in basal RPMI medium (Life Technologies #11875093) supplemented with B27 (Life Technologies #17504044, Carlsbad, CA, USA), Y-27632 ROCK inhibitor (20 µM; Tocris #1254) and 10% foetal bovine serum (Sigma-Aldrich). The medium was changed after 24 h to RPMI/B27 medium.

### 2.6. hPSC-CMs Viability and Immunostaining

Viability studies were performed in gels with different GelMa concentrations (5 and 10 wt. %) and different MWCNTs concentrations of 0.5 and 1 mg mL^−1^. hPSC-CM were washed in complete DPBS and incubated for 30 min in 1 µM acetoxymethyl (AM) calcein solution (Sigma Aldrich #C1359) in DPBS to stain viable cells and dead cells were stained with 5 µM ethidium homodimer I (Sigma Aldrich #E1903) in PBS. Fluorescence images were taken on an inverted EVOS FL Fluorescence microscope. Statistical significance was calculated using a *t*-test (N = 3, *n* = 2; ±SD)

Immunostaining of hPSC-CMs was performed by fixing cells in 2% formaldehyde (VWR) and permeabilised with 0.1% Triton-X 100 (Sigma-Aldrich). Non-specific binding was blocked with 4% foetal bovine serum (FBS) (Sigma-Aldrich) in DPBS for 1 h. Samples were immunostained with a monoclonal primary antibody against sarcomeric alpha-actinin produced in mouse (Abcam #ab9465, Cambridge, UK) at a concentration of 1:400 in DPBS and incubated overnight at 4 °C. A solution of 0.05% of Tween 20 was used to wash the samples and a solution of goat anti-mouse secondary antibody IgG was added (1:1000; Abcam # ab6785) and incubated for 2 h. Samples were washed with a washing solution and exposed to Hoechst 33258 (5 µg mL^−1^; Sigma Aldrich) and phalloidin (1:150, Thermo Fischer Scientific) for 2 h. Fluorescence images were taken on an inverted EVOS FL Fluorescence microscope.

### 2.7. Two-Photon Polymerisation of Hydrogels

A Nanoscribe Photonic Professional GT fitted with a fibre laser was used for the fabrication following similar protocols developed previously [27]. The wavelength of the laser is 780 nm with a pulse frequency of 80 MHz and a pulse duration of 120 fs. An oil immersion objective was used for the printing (63×, NA = 1.4, WD = 190 µm). The laser power used for the work varied from 30 to 100 mW and the scan speed varied from 500 to 50,000 µm/s for printing condition optimisation. The whole printing and developing process was carried out in a UV-free environment.

Briefly, 0.5 wt% of the photoinitiator P2CK was added to MWCNTs-GelMa and dissolved by magnetic stirring for 15 min before printing in a water bath (40 °C). Droplets of GelMa and MWCNTs-GelMa inks were placed in glass coverslips previously cleaned with ethanol and dried. These were then placed in the equipment printing stage. The optimised process condition used for printing pristine GelMa was 90 mW laser power and 10,000 µm/s print speed. The hatch spacing was 0.2 and 0.4 µm layer thickness. For the MWCNTs-GelMa, the print parameters were 40 mW laser power, 10,000 µm/s print speed and 0.2 and 0.3 µm slicing. The laser power for MWCNTs containing GelMa had to be significantly reduced to prevent boiling of the material due to high laser energy absorption and conduction within the material.

After fabrication of the different structures, excess material was gently removed by immersing the coverslip in a beaker containing DPBS maintained at 40 °C. Upon developing, the samples were stored in DPBS to prevent dehydration. Printed samples were wet-imaged using an optical microscope with the sample immersed in DPBS. For SEM imaging, a Hitachi TableTop SEM was used. Before imaging, the sample was dehydrated by freeze drying.

## 3. Results and Discussion

**Figure 1:**
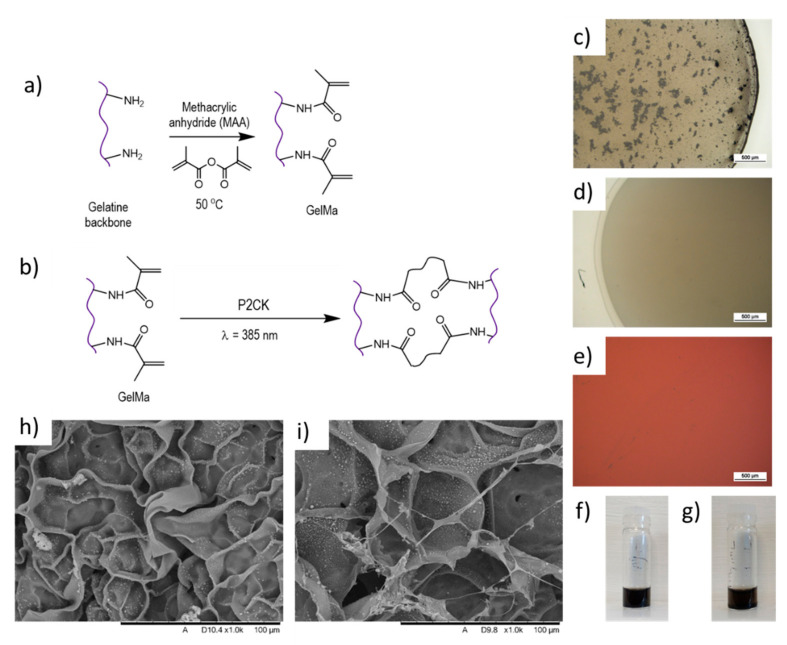
Mechanisms of (**a**) modification of gelatine with methacrylate groups to obtain gelatine methacrylate (GelMa) and (**b**) photo-polymerisation of GelMa groups to obtain a cross-linked gel. GelMa pre-polymer hydrogel (5 wt. %) with 1 mg mL^−1^ multi-walled carbon nanotubes (MWCNTs-GelMa) (**c**) before and (**d**) after centrifugation and (**e**) ink formulation after addition of photoinitiator. Stability of MWCNTs-GelMa solutions (**f**) 3 and (**g**) 45 h after MWCNTs dispersion. SEM imaging of lyophilised (**h**) GelMa and (**i**) MWCNTs-GelMa structures.

Gelatine was chemically modified using MAA in order to introduce methacrylic groups to the gelatine backbone with the potential to be UV-cross-linked. MAA binds to amine groups present in the amino acid lysine (Lys) (Figure 1a). Once this reaction was completed, solutions were dialysed to eliminate unreacted MAA. To induce the photo-cross-linking of the GelMa, P2CK is added to the solution, which then gelates the material after exposure with the 2PP laser (Figure 1b).

The degree of conversion/methacrylation was then calculated from the lysine methylene peak of gelatine and the GelMa to confirm the presence of methacrylate groups in the gelatine. It is expected that the methylene lysine proton peak intensity would decrease with an increase in methacrylation and an increase in methacrylamide, with respect to conversion. The results (Figure S1) show a clear indication of the decrease in lysine groups and an increase in acrylic proton. This was evident from two individual batches of GelMa prepared using this protocol. Upon calculating the degree of conversion, GelMa batch 1 (GB1) showed 86.9% and GelMa batch 2 (GB2) showed 88.4% conversion.

To increase the conductivity of GelMa, 1 mg mL^−1^ MWCNTs were used as conductive nano-fillers. Initial experiments were performed to compare the dispersion of MWCNT functionalised with carboxylic groups (-COOH) to those not functionalised. It became apparent that without functionalisation, the MWCNTs aggregated and were difficult to disperse in the GelMa (Figure S2) even with several hours of ultrasonication. These larger aggregates in the order of hundreds of microns can prevent the nano-structures to be fabricated in 2PP as the energy absorption is non-uniform, which can affect the polymerisation. A similar effect has also been reported in single-walled carbon nanotubes (SWCNTs), where aggregation and sedimentation were observed 3 h after ink preparation [30]. This study also indicated a direct correlation between SWCNTs concentration used and the aggregation effects.

Although aggregates were also observed with -COOH functionalised MWCNTs (Figure 1c), a uniform dispersion was achieved after ultrasonication for 15 min in an ice bath (to prevent heating-up of the material) and centrifugation at 6000 rpm for 30 min at 40 °C (to prevent gelation). Centrifuging resulted in a clear solution, ideal for subsequent fabrication of three-dimensional structures using the two-photon polymerisation technique due to its improved dispersion and homogenous distribution of the material, which affects its performance. -COOH functionalised MWCNTs were then selected for subsequent dispersion in GelMa and ink formulation (MWCNTs-GelMa). To complete the ink formulation for photopolymerisation, P2CK was added to the MWCNTs-GelMa solution. No further aggregation was noted upon adding the photoinitiator (Figure 1e).

The dispersion stability of MWCNTs-GelMa solutions was investigated to analyse whether this was adequate for 2PP fabrication by leaving the dispersion stationary after sonication and centrifugation. No sedimentation was observed in MWCNTs-GelMa solutions after 3 and 45 h of the dispersion of MWCNTs (Figure 1f,g). This allows sufficient time for 2PP fabrication of the structures and indicates that sedimentation of MWCNTs should not occur during the manufacturing of the 2PP structures. Conversely, the composition containing non-functionalised MWCNTs started to settle within an hour for the 1 mg.mL^−1^ composition (Figure S3).

Lyophilised samples of GelMa and MWCNTs-GelMa (Figure 1h,i) were obtained, where MWCNTs can be seen forming an interconnecting network inside GelMa pores. We hypothesise that this demonstrates the ability of MWCNTs to form an interconnected conductive mesh that contributes to increased conductivity.

**Figure 2:**
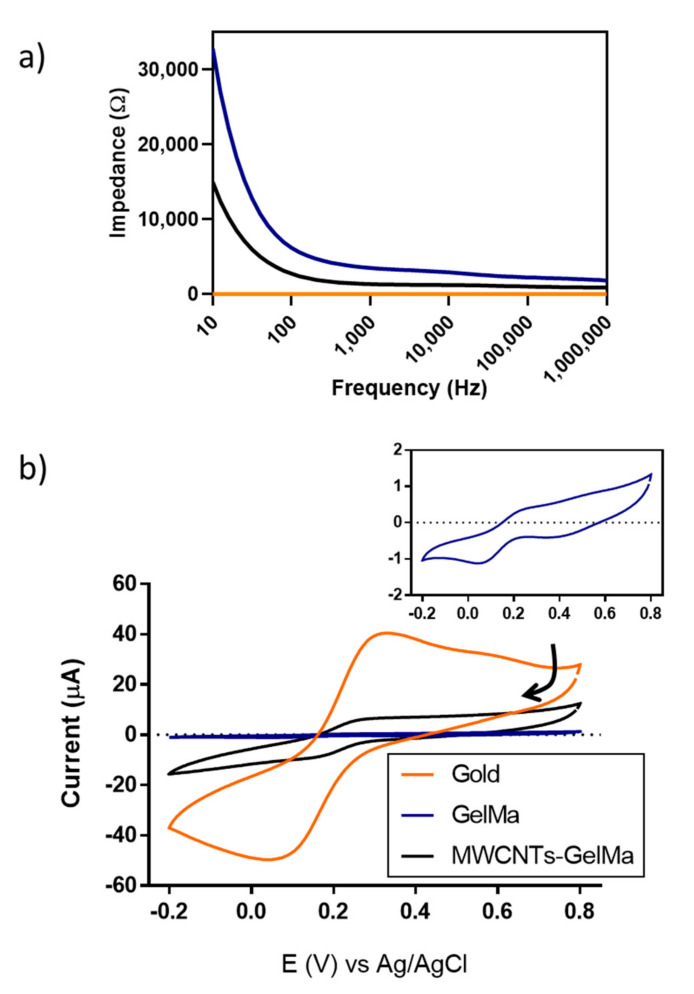
Electrochemical characterisation of gels. (**a**) Impedance spectroscopy measurements of GelMa and MWCNTs-GelMa (1 mg mL^−1^) compared to gold surfaces. (**b**) Typical cyclic voltammograms (CVs) of gels developed in situ on gold surfaces. Potassium hexacyanoferrate (II) (1 mM) in phosphate buffer saline (PBS) was used as an electrolyte. CVs were measured from 0.8 to −0.2 at 100 mV s^−1^ vs. an Ag/AgCl reference electrode. Arrow indicates the direction of the CV. All measurements were performed in triplicate. Inset CV corresponds to GelMa, shown for clarity.

To confirm this, the electrochemical activity of the hydrogels was studied using electrochemical impedance spectroscopy and cyclic voltammetry (CV) to determine the conductivity and ability to exchange electrons of the MWCNT-GelMa. Electrochemical characterisation was performed on non-2PP printed structures, as the micron range dimensions (100 µm × 100 µm × 10 µm) of the actual printed structures is not characterizable with the systems used. Significant differences in the impedance values of the different materials can be seen in Figure 2a. GelMa was the most resistive material as expected, as collagen molecules typically present resistance values in the order of MΩ [33]. Suspension of MWCNTs into hydrogels decreased the impedance of the materials, which depended on the concentration of MWCNTs. This effect was more evident at lower frequencies (10–100 Hz) as materials behave as capacitors, where impedance values are inversely proportional to frequency. To compare our soft conductive hydrogels to a more traditional conductive material, the impedance of bare gold was also measured, with the lowest impedance of 4.8 Ω. From this, it can be seen that MWCNT-modified hydrogels have intermediate values of impedance when compared to highly conductive materials (gold) and resistive materials (GelMa). For instance, at 100 Hz, the impedance of MWCNTs-GelMa corresponds to 2745.06 Ω. This contrasts the values of pristine GelMa (6209.67 Ω) and bare gold (4.8 Ω).

Cyclic voltammograms (CVs) of GelMa, MWCNTs-GelMa and gold in the presence of the well-known redox-active molecule potassium hexacyanoferrate (II) can be seen in Figure 2b. On bare gold, a reduction peak corresponding to gold was detected at 0.436 V. This peak was more evident in the absence of the redox-active molecule (Figure S4). Peaks corresponding to reduction and oxidation of potassium hexacyanoferrate (II), respectively, were found at 0.043 and 0.322 V. These peaks were also detected in the presence of GelMa at 0.044 and 0.286 V and MWCNTs-GelMa at 0.153 and 0.271 V. It is important to note that the lowest peak separation corresponded to MWCNTs-GelMa (0.118 vs. 0.279 V in gold and 0.242 V in GelMa). Peak separation can be used to determine the electrochemical reversibility of a system. Lower values of peak separation can be interpreted as an increase in the electrochemical reversibility, where electrochemical reversible systems possess a value of peak separation of 0.059 V [34]. This could be due to the ability of the material to exchange electrons more easily. A gold peak can also be seen in the CVs of GelMa and MWCNTs-GelMa at 0.400 V as gels were formed in situ on gold electrodes before performing the measurements (Figure 2b and Figure S4). The peak height of CVs can be related to the number of electrons exchanged, being higher at the gold surfaces followed by MWCNTs-GelMa and GelMa. It can be concluded that the addition of MWCNTs to GelMa increases the ability to exchange electrons within the hydrogel.

The next part of the investigation was aimed at determining whether GelMa and MWCNTs-GelMa could be used as a potential material in 2PP in the manufacturing of biomimetic scaffolds. To do this, materials were incubated with human pluripotent stem cell-derived cardiomyocytes (hPSC-CMs), after which the viability and structural morphology of the cells were studied. Details of the hPSC-CMs and differentiation protocols used can be found elsewhere [32].

**Figure 3:**
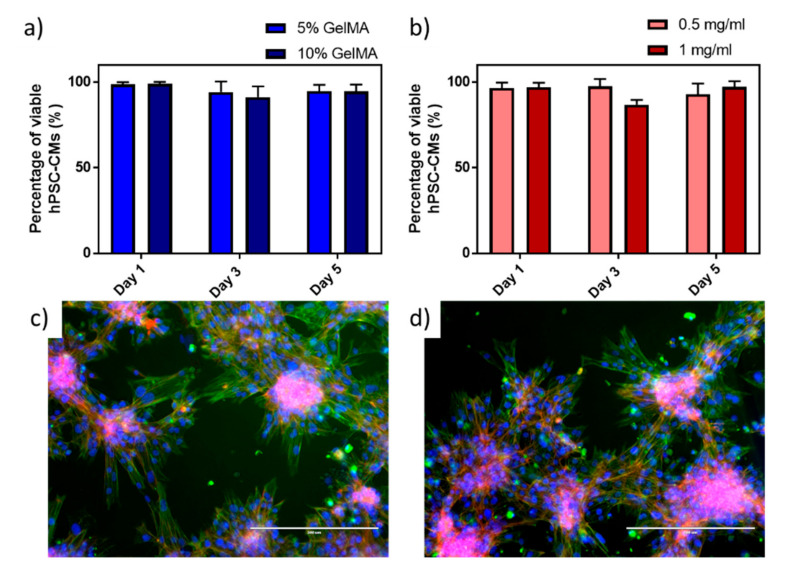
Viability studies performed on human pluripotent stem cell-derived cardiomyocytes (hPSC-CMs) on (**a**) 5% and 10% GelMa and (**b**) 0.5 mm mL^−1^ and 1 mg mL^−1^ MWCNTs in 5% GelMa. Fluorescence images of immunostained hPSC-CMs after 7 days of culture in (**c**) 5% GelMa and (**d**) 1 mg mL^−1^ MWCNTs in 5% GelMa. Nuclei were stained with a Hoechst probe (blue), actin fibres were stained with a phalloidin probe (red), and sarcomeres were immunostained with anti-alpha actinin (green). Scale bar 200 µm.

A live/dead fluorescence staining (Figure S5) was performed on hPSC-CMs at different GelMa concentrations (5 and 10 wt.%), to identify the optimal concentrations that support cell viability. hPSC-CMs were incubated on GelMa structures for 5 days, where no significant differences were found between concentrations of GelMa with viabilities above 90% (Figure 3a). The lowest concentration of GelMa (5%) was selected in the following experiments, as higher concentrations increase the viscosity of the solutions, thus affecting the dispersion of MWCNTs. Effects of MWCNTs on cell viability were also studied at different MWCNTs concentrations of 0.5 and 1 mg mL^−1^ since certain cytotoxic effects can be associated with MWCNTs [35]. However, as MWCNTs were confined in the hydrogels, viability values were above 86% in all the cases (Figure 3b) and there were no significant differences with the viability of hPSC-CMs in GelMa without MWCNTs. This confirms previous reported results where 90% cardiomyocyte viability was achieved at 3 and 4 mg mL^−1^ MWCNTs concentration in GelMa [36].

Morphology of hPSC-CMs was assessed by fluorescence microscopy of intracellular structures on GelMa (Figure 3c) and MWCNTs-GelMa (Figure 3d) materials. As can be seen from the images, hPSCM-CMs tend to aggregate in individual clusters of cells. On the edges of the cellular agglomerations, it is possible to see elongated hPSCM-CMs with visible sarcomeres. No differences in cell morphology can be observed when using different materials.

Once it was demonstrated that materials support cell viability and development, optimisation of printing parameters was carried out. Initial experiments were performed with the pristine GelMa to find the optimal process conditions. Figure S6 shows the screenshots taken during the fabrication process of an ultra-thin film of GelMa (100 × 100 × 10 µm). The bright line in the image indicates the laser scanning the sample. At the start of the optimisation process, some material was being polymerised but structures did not adhere to the glass coverslips. Some burning and boiling effects were also observed, and black bubbles indicated an excess of heat/energy. Upon optimisation, it can be seen that a uniform structure can be obtained. Some of the values used in the optimisation process can be seen in Figure S7. It should be noted that printing hydrogels using 2PP is more complex than printing non-hydrogels. One of the challenges of 2PP printing of hydrogels is the drying of the ink droplet with time as shown in Figure S8. Materials need to be processed before water evaporates to avoid dehydration of the material and destruction of the structures, therefore, manufacturing of bigger constructs is limited with the current technology. The optimised process condition in this study to print pristine GelMa with 0.5% photoinitiator concentration was 90 mW laser power and 10,000 µm/s print speed. The hatch spacing was 0.2 and 0.4 µm layer thickness.

**Figure 4:**
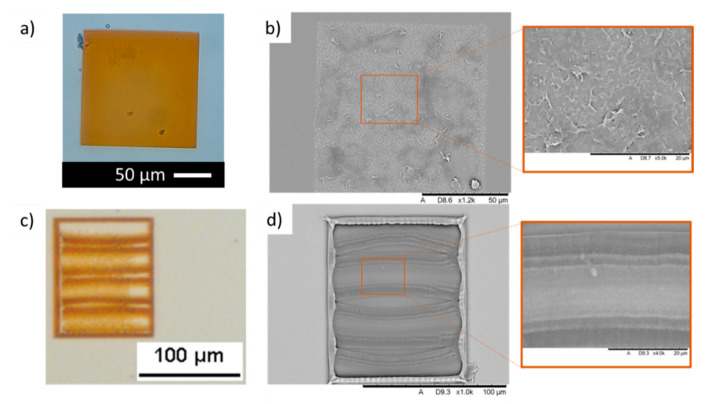
Hydrogel GelMa structures developed by 2PP. (**a**) Optical microscopy and (**b**) SEM imaging of hydrated and dehydrated GelMa ultra-thin films, respectively. (**c**) Optical microscopy and (**d**) SEM imaging of 120 µm × 100 µm complex GelMa scaffolds with 10 µm channels developed by 2PP.

These optimal conditions were used to print a sample scaffold structure as shown in Figure 4a. The optical microscopic image was taken by soaking the sample in DPBS to prevent dehydration of the material. Hydrogels swelling can be seen as the printed material was left soaking in DPBS for a few hours before characterisation. In addition, slight damages might have occurred during the developing state where un-reacted material is removed from the structures. Since the concentration of GelMa is only 5% in this study, structures are highly prone to damage. However, the lower concentration of GelMa prevents the drying of the ink droplet during the 2PP process and demonstrated good MWCNTs dispersion. The equipment used in this study does not have any additional modification to prevent the drying of the material. This is important to consider while performing further developments. An SEM image of the dehydrated structure has also been shown to showcase the success of printing GelMa in 2PP (Figure 4b).

The 2PP capabilities of printing resolution in the micro and nanoscale allow us to introduce further complexity to our structures, which is key in developing scaffolds for tissue engineering and regenerative medicine applications. To test this, 120 µm × 100 µm scaffolds were manufactured including three 10 µm channels (Figure 4c). Despite some deformation that took place during material development and lyophilisation, the different elements of the structure could also be identified by SEM imaging (Figure 4d).

During the MWCNTs-GelMa printing optimisation, a higher concentration of MWCNTs was used to test the printing capabilities of the material. A concentration of 3 mg mL^−1^ MWCNTs was initially used. This ink proved challenging to print for two main reasons. First, the intense black colour of the material made it difficult to observe the printing area. Second, the MWCNTs absorbed significant laser energy resulting in localised boiling during the printing process that disrupted the manufacturing. Hence this MWCNTs concentration was no longer considered.

**Figure 5:**
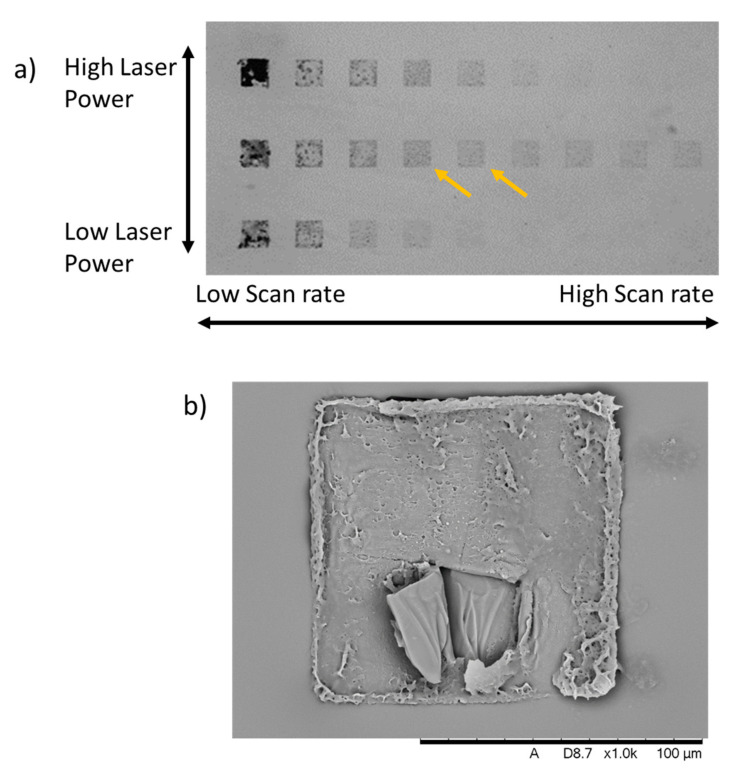
(**a**) Optimisation conditions of MWCNTs-GelMa inks to produced thin-films by 2PP. Laser power was varied between 50, 40 and 30 mW and the scan rate was varied between 500, 1000, 2000, 5000, 10,000, 20,000, 30,000, 40,000 and 50,000 µm/s. Layer distance corresponds to 0.3 µm. Hatch spacing corresponds to 0.2 µm. Yellow arrow indicates our selected optimal conditions. (**b**) SEM image of dehydrated MWCNTs-GelMa thin-film manufactured by 2PP.

The 1 mg mL^−1^ concentration of MWCNTs-GelMa enabled significantly more straightforward printing as fewer boiling events were observed during the printing process, possibly due to the lower concentration of MWCNTs. However, it was difficult to visualise the printing process and the laser scanning over the material. Hence the printing conditions were pre-programmed with varied layer thickness, hatch distance, laser power and scan speed. This is shown in Figure 5a, where thin films (layer thickness 0.3 µm) were imaged by optical microscopy after printing and material development.

At lower scan rates, the material causes burns as indicated by the black appearance of the structures and this effect is particularly evident with increased laser power. This is probably due to the increased exposure of the material to the laser energy. Similar effects were also described on graphene oxide composites, attributed to localised excessive heat generated during a high rate of photothermal conversion under the laser radiation [37]. Conversely, higher scan rates lead to the structures not being formed. These effects were also observed at different layer thicknesses (0.2 and 0.4 µm) (Figure S9). Printing parameters of 40 mW laser power, 5000–10,000 µm/s print speed, 0.3 µm layer thickness and 0.2 µm hatch spacing rendered more stable structures.

It can be noted that the energy required for printing the MWCNTs-GelMa structures (40 mW) was significantly lower than the energy required to print the pristine GelMa structures (90 mW). This requirement for a lower energy density is mainly due to the material’s properties, as increased energy absorption and conduction within the material can lead to overheating and boiling. Spatial resolution of MWCNTs composites have also been compared to pristine materials, where higher resolutions were achieved in the composites structures [30]. This could be due to the lower printing energy used as this study reported that lower laser intensities led to higher printing resolution.

SEM imaging of the dehydrated MWCNTs-GelMa structures (Figure 5b) shows that microstructures of this material can be manufactured. Some structural damage can be observed due to the low concentration of GelMa that makes structures extremely fragile and prone to damage during the cleaning, dehydration and sputter coating processes. This can be prevented in the future by increasing the adhesion of the structures to the glass coverslips through a silanisation process, creating covalent bonds between the structures and the substrate. Moving forward, modifications to the 2PP equipment that could allow the printing of larger structures through reduced water evaporation rates from inks could offer a significant advancement in the development of more complex conductive structures for bioelectronics and electronics applications.

## 4. Conclusions

In this study, we have presented a strategy of the manufacture of conductive hydrogel nano-/microstructures by 2PP for potential applications in bioelectronics. Conductive hydrogel inks (MWCNTs-GelMa) consisted of three components: (i) GelMa, as the main hydrogel component, (ii) a dispersion of -COOH functionalised MWCNTs, acting as conductive nanofillers; (iii) P2CK, as a water-soluble biocompatible photoinitiator. Electrical properties of the hydrogels were enhanced after the addition of the MWCNTs, showing lower impedance values and improved ability to exchange electrons compared to pristine GelMa. This material was also shown to support hPSC-CMs viability. Films with a thickness of 10 µm and scaffolds were fabricated using 2PP, where the optimal printing parameters corresponded to 40 mW laser power, 5000–10,000 µm/s print speed, 0.2 µm hatch spacing and 0.3 µm layer thickness. While further technological developments are necessary for the current embodiment to prevent ink dehydration during the printing process to fabricate more complex structures, this work opens up the opportunity to fabricate conductive biomimetic scaffolds in extremely low resolution using hydrogels.

## Data Availability

Data supporting results can be found at the University of Nottingham repository (https://nottingham-repository.worktribe.com/).

## Supplementary Materials

The following are available online at https://www.mdpi.com/article/10.3390/polym13071038/s1, Figure S1: ^1^H NMR spectra of (a) gelatine and two different batches of gelatine methacrylate (GelMa), (b) GB1 and (c) GB2; Figure S2: (a) 5× and (b) 50× optical microscopy images of the GelMa after addition of multi-walled carbon nanotubes (MWCNTs) without cross-functionalisation; Figure S3: GelMa dispersions including 1 mg mL^−1^ of unfunctionalized MWCNTs after 0 (left) and 60 (right) min. Yellow arrow indicates the location of the deposits that lead to an uneven distribution of MWCNTs in the dispersion; Figure S4: Cyclic voltammograms (CVs) of gels developed in situ on gold surfaces using PBS as electrolyte. CVs were measured from 0.8 to −0.2 at 100 mV s^−1^ vs. an Ag/AgCl reference electrode (*n* = 3). Arrow indicates direction of the CV; Figure S5: Fluorescence images of hPSC-CMs after live/dead staining with ethidium homodimer 1 (dead cells) and calcein-am (viable cells) on (a) 5% GelMa, (b) 10% GelMa, (c) 5% GelMa modified with 0.5 mg mL^−1^ MWCNTs and (d) 5% GelMa modified with 1 mg mL^−1^ MWCNTs on days (i) one, (ii) three and (iii) five of incubation. Scale bar 400 µm; Figure S6: Screenshots taken during 2PP printing of GelMa ultra-thin films. Bright line shows laser photopolymerising GelMa structures and black dots indicate bubbles from burnt areas due to an excess of heat. Optimal conditions were used to print GelMa film in the lower right; Figure S7: Optical microscopy images of GelMa films and some of the parameters used during the optimisation; Figure S8: Printing stage of the 2PP showing the effect of water evaporation 60 min of addition of the ink droplet. Red line indicates the original area occupied by the ink; Figure S9: Optimisation conditions of MWCNTs-GelMa inks to produced thin-films by 2PP. Laser power was varied between 50, 40 and 30 mW and scan rate was varied between 500, 1000, 2000, 5000, 10,000, 20,000, 30,000, 40,000 and 50,000 µm/s. Layer thickness corresponds to (a) 0.4 and (b) 0.2 µm. Hatch spacing was kept constant at 0.2 µm.
