# Supplementary material for: Development of Conductive Gelatine-Methacrylate Inks for Two-Photon Polymerisation
Source: Polymers (Basel). 2021 Mar 26;13(7):1038. doi: 10.3390/polym13071038 (PMC8037899; doi:10.3390/polym13071038)
Supplement: Supplementary file 1 [file polymers-13-01038-s001.pdf]

polymers

MDPI

Supplementary information and figures

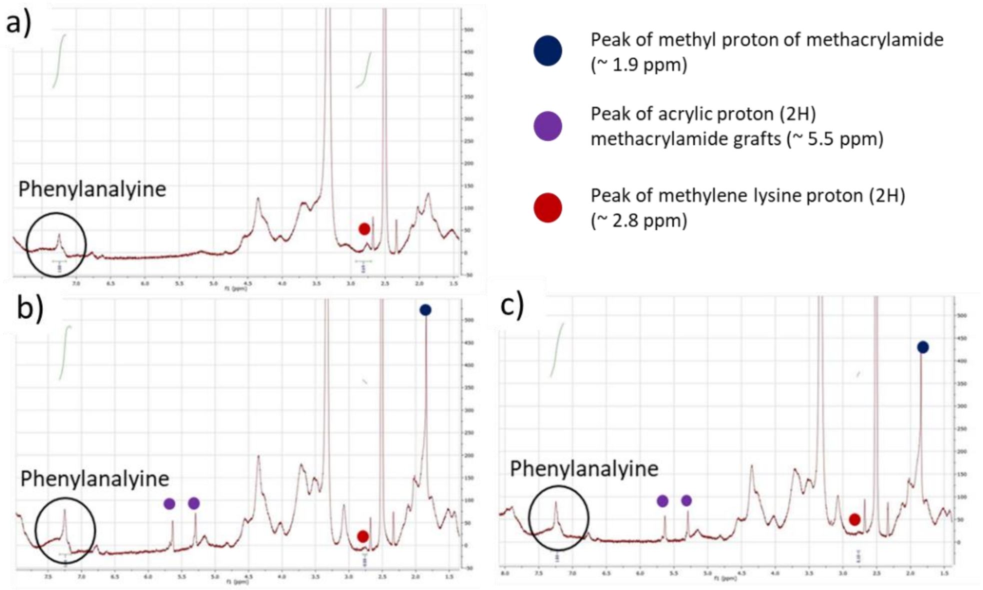

Figure S1. ¹H NMR spectra of (a) gelatine and two different batches of GelMa, (b) GB1 and (c) GB2.

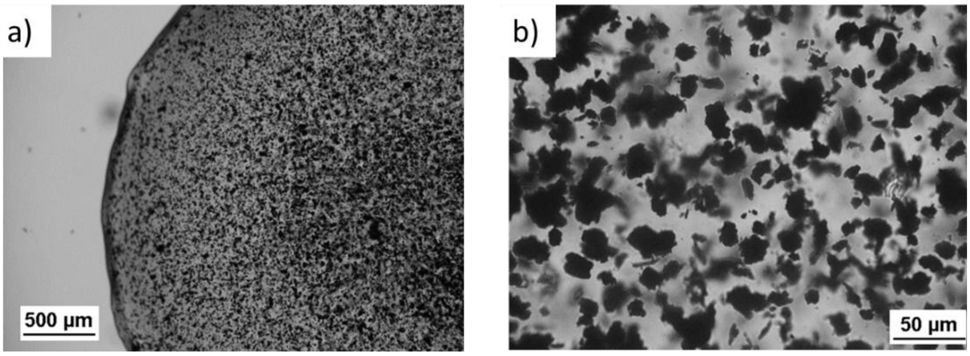

Figure S2. (a) 5x and (b) 50x optical microscopy images of the GelMa after addition of MWCNTs without cross functionalisation.

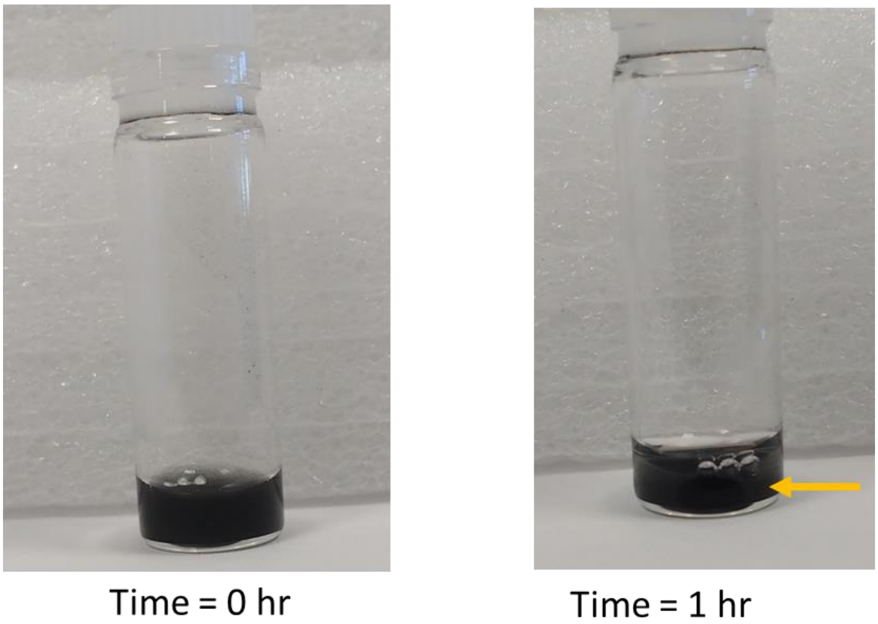

Figure S3. GelMa dispersions including 1 mg mL^{-1} of unfunctionalized MWCNTs after 0 (left) and 60 (right) minutes. Yellow arrow indicates the location of the deposits that lead to an uneven distribution of MWCNTs in the dispersion.

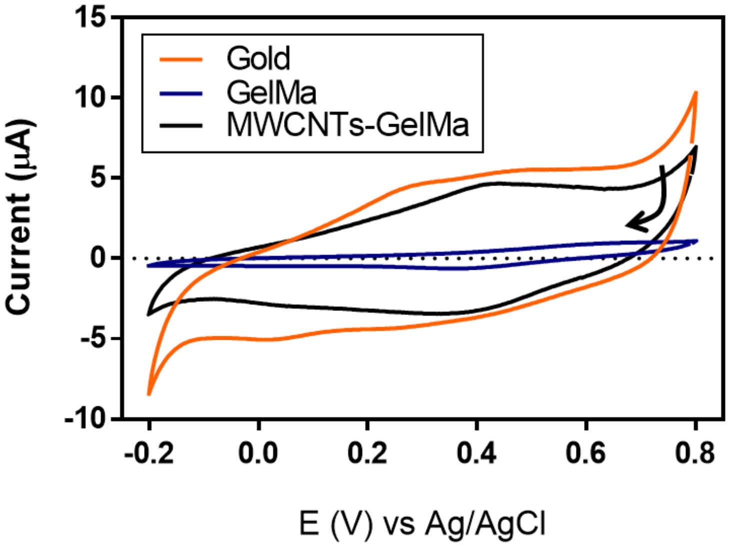

Figure S4. Cyclic voltammograms (CVs) of gels developed in situ on gold surfaces using PBS as electrolyte. CVs were measured from 0.8 to -0.2 at 100 mV s-1 vs an Ag/AgCl reference electrode (n=3). Arrow indicates direction of the CV.

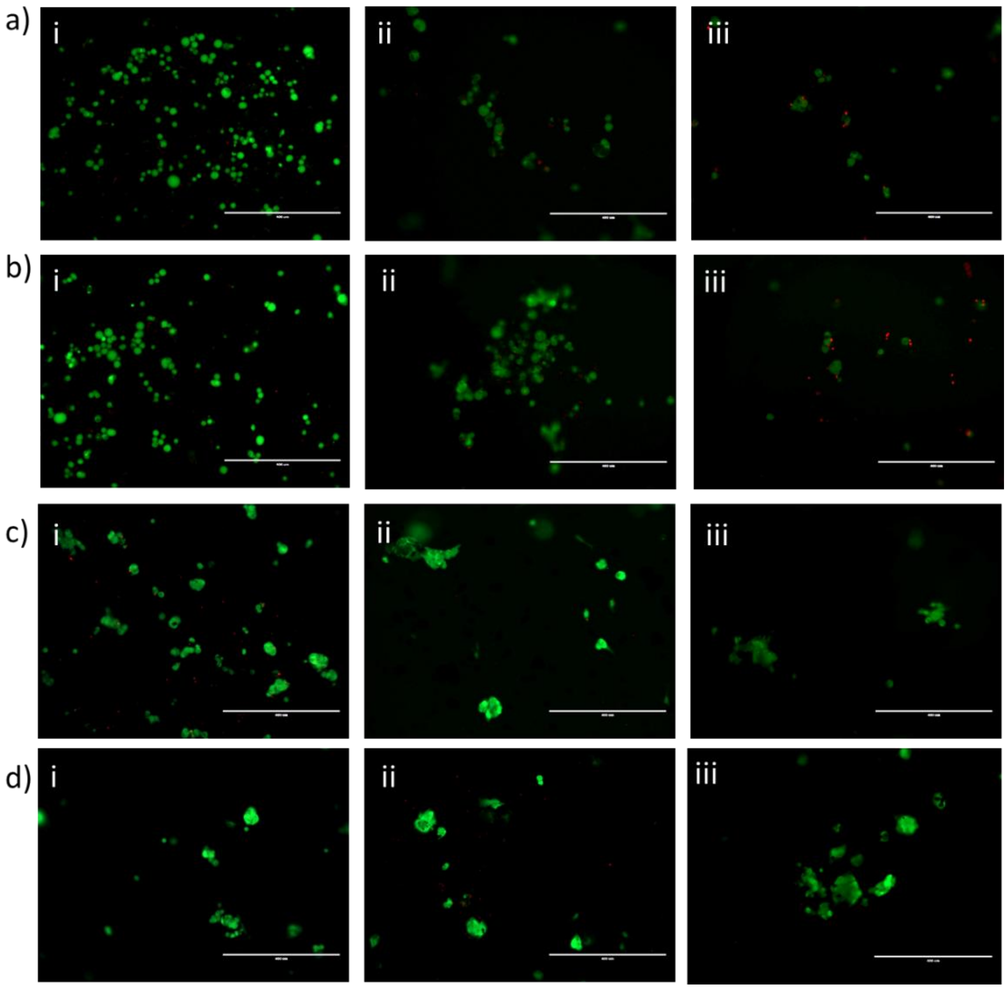

Figure S5. Fluorescence images of hPSC-CMs after live/dead staining with ethidum homodimer 1 (dead cells) and calcein-am (viable cells) on a) 5% GelMa, b) 10% GelMa, c) 5% GelMa modified with 0.5 mg ml^{-1} MWCNTs and d) 5% GelMa modified with 1 mg ml^{-1} MWCNTs on days (i) one, (ii) three and (iii) five of incubation. Scale bar 400 µm.

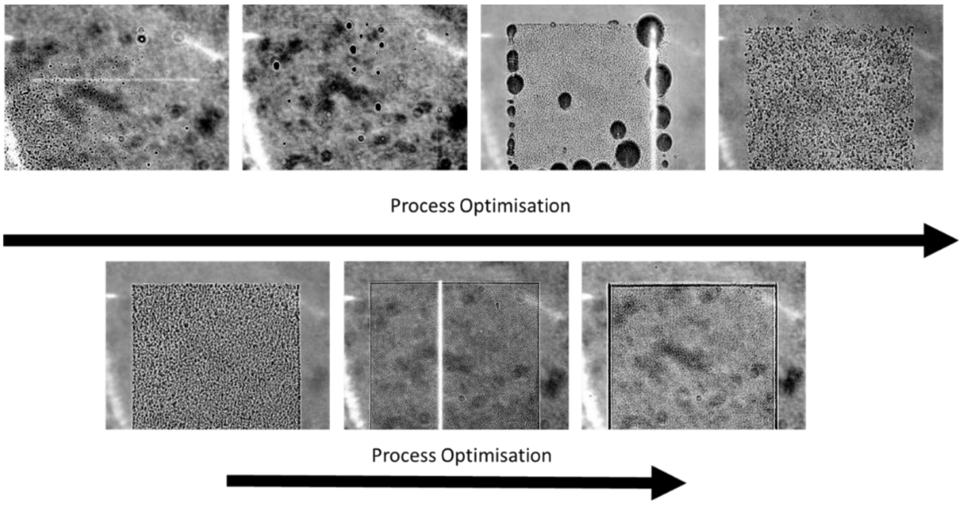

Figure S6. Screenshots taken during 2PP printing of GelMa ultra-thin films. Bright line shows laser photopolymerising GelMa structures and black dots indicate bubbles from burnt areas due to an excess of heat. Optimal conditions were used to print GelMa film in the lower right.

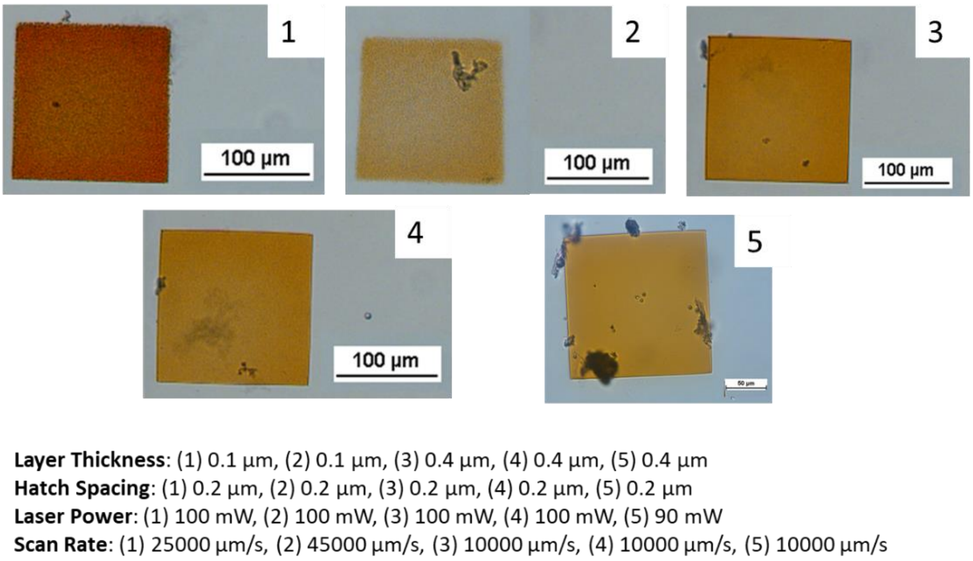

Figure S7. Optical microscopy images of GelMa films and some of the parameters used during the optimisation

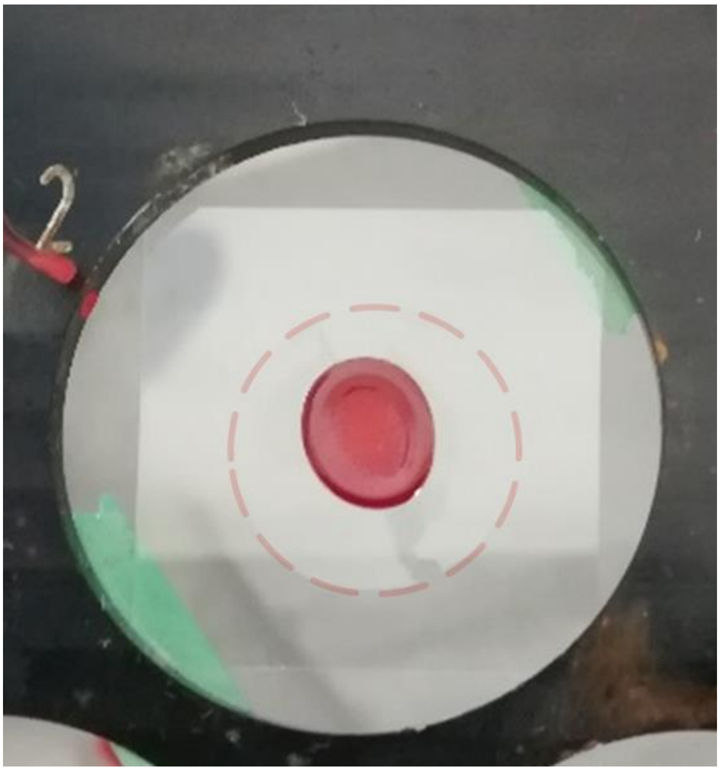

Figure S8. Printing stage of the 2PP showing the effect of water evaporation 60 minutes of addition of the ink droplet. Red line indicates the original area occupied by the ink.

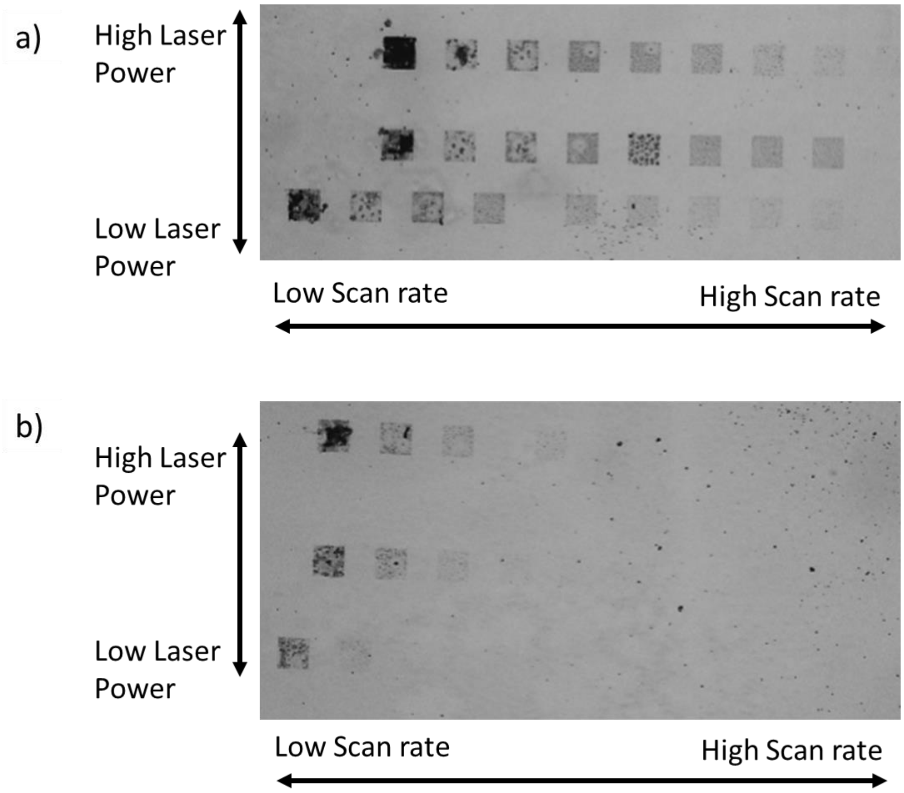

Figure S9. Optimisation conditions of MWCNTs-GelMa inks to produced thin-films by 2PP. Laser power was varied between 50, 40 and 30 mW and scan rate was varied between 500, 1000, 2000, 5000, 10000, 20000, 30000, 40000 and 5000 µm/s. Layer thickness corresponds to (a) 0.4 µm and (b) 0.2 µm. Hatch spacing was kept constant at 0.2 µm.
